# COVID‐19, cancer post‐pandemic risk, and the radiation oncology physicist

**DOI:** 10.1002/acm2.13628

**Published:** 2022-05-08

**Authors:** Mary Beth Allen

**Affiliations:** ^1^ School of Public Health University of Louisville Louisville Kentucky USA

**Keywords:** cancer screening, cancer survival, COVID‐19 and cancer, long COVID, medical physicists, radiation oncology

## 1 INTRODUCTION

The COVID‐19 pandemic has had a profound impact on the health‐care industry in the United States and worldwide. Nearly every aspect of healthcare was impacted, including but not limited to research and development, purchasing and inventory management, health‐care delivery, human resource management, and standard operating procedures. In almost every care setting, artifacts of the COVID‐19 pandemic linger in the form of changes in policies and procedures, redistribution of staff and resources, shortages and oversupplies of key resources, and a shared desire to capture consistency and normalcy following a 24‐month storm. In the United States, the sustainability of health‐care systems following this major disruption remains an important challenge as revenue from profitable elective outpatient procedures shrunk and the demand for critical care soared. Furthermore, multiple surges in the pandemic required a shift in resources and staff to less profitable intensive care units, whereas regions impacted most triggered a national redistribution of resources, nurses, and other health‐care workers.

The health‐care industry in the United States is complicated. Although some market forces like competition and quality impact health‐care delivery in a similar manner to other private sector industries, health systems also coordinate and collaborate to provide population health services, deliver individual care, participate in the advancement of knowledge, and establish processes and standards of care based on data. The advancement and implementation of knowledge and best practices is a shared experience based on the evidence, debate, and consensus. Given the specialized care provided by radiation oncology physicists, participation in the health‐care market has been characterized more as collaborative than competitive. Despite market forces and disruptions, these physicists have remained focused and responsible for designing, delivering, and reporting the outcome of the treatment planning and delivery process.

Population health continues to intersect with health‐care delivery. Prior to COVID‐19, the introduction of value‐based reimbursement models facilitated a shift in risk from health plans to providers. Instead of incentivizing providers to maximize the number of interactions, this shift tied reimbursement to outcomes and encouraged health systems to engage in population health. Many providers were suddenly expected to assume a larger role, including medical physicists being asked to assume the role in some care settings as biomedical scientists to defend radiation oncology outcomes.

COVID‐19 has complicated both the entry and outcome of the radiation oncology intervention. Cancer screening rates dipped during the pandemic and have yet to recover. Active cancer with concomitant COVID‐19 infections continues to represent a new and potentially deleterious risk for cancer patients. COVID‐19 prevention methods continue to accumulate, which can be especially beneficial for high‐risk patients under care. Also challenging, the implications for the future of cancer care are not well understood given the persistence of the COVID‐19 pandemic continuing to cause new and recurring infections as well as the potential increased risk of cancer and other complications in patients recovering from COVID‐19 infection. Finally, long COVID is greatly complicating the posttreatment evaluation, progression, and outcome of cancer patient survival and quality of life.

## 2 SCREENING

Early identification is perhaps the most effective intervention in cancer care. For this reason, countless screening tests are recommended for at‐risk populations due to age, family history, genetic testing, or personal medical history as part of standard care. Current recommended screening tests have been validated by significant evidence that early detection reduces health‐care costs, improves clinical outcomes, and prevents early mortality. The key to the effectiveness of screening programs, however, is participation. The COVID‐19 pandemic is associated with a sharp decline in screening. According to one report, screening for breast cancer declined by 90.8% representing 3.9 million fewer tests among the years 2018, 2019, and 2020 in patients without active cancer. Colorectal cancer also decreased during the same time period and in the same population by 79.3%, which means 3.8 million fewer screening tests were administered. Finally, screening for prostate cancer decreased by 86.7% with 1.6 million fewer tests performed. The impact of screening participation was not equally distributed in the United States with regional differences in both declines in screening and speed to recovery. Patients with a higher socioeconomic status represented a sharper decline, but participation in telehealth reinforced adherence to screening.^1^ A British study noted a 24% decrease in cancer screening recorded in primary care centers following a study of more than 8000 patients from over 600 care facilities. However, urgent referrals only decreased by around 10.5% indicating a low impact of primary care providers coordinating care in the event of cancer detection.^2^ As of 2022, cancer screening rates have not recovered in the United States. Increasing cancer screening to pre‐pandemic levels is a priority of the American Cancer Society's National Consortium for Cancer Screening and Care (ACS National Consortium) according to a recent announcement.^3^ Another notable challenge in cancer screening is the occurrence of subclinical unilateral axillary lymphadenopathy following recent vaccination, which can have the appearance of malignancy on mammography. This has led to recommendations regarding the timing of vaccination in relation to cancer screening.^4^

## 3 TREATMENT

Risk stratification for severe COVID‐19 infection has proved challenging for care providers. Following exposure and the onset of symptoms, patients were routinely instructed to remain quarantined and self‐manage symptoms up until the need for hospitalization, as no outpatient therapies were proven effective. This was the case for all risk groups, but it was generally advised that older adults with comorbid conditions were at a higher risk for hospitalization and mortality. Although on the population level, hospitalization and mortality rates are disproportionately observable in traditionally high‐risk populations, major disparities in outcomes within both high‐ and low‐risk populations made risk calculation for all populations less predictable. The confounding occurrence of asymptomatic disease in all risk groups just added to the confusion.

Several studies show an association between cancer and poor clinical outcomes. A study of nearly 7000 patients hospitalized in New York between 16 March and 31 July 2020 found active cancer was not associated with an increased risk of ICU admission but was associated with a higher risk of all‐cause mortality. Survival was more strongly associated with patients with a history of cancer as opposed to active cancer.^5^ Another study of over 4000 hospitalized patients in New York echoed these findings.^6^ A British study examined active cancer alongside other comorbidities, including diabetes mellitus, hypertension, coronary artery disease, and dyslipidemia, finding a death rate in active cancer patients to be nearly double.^7^

For cancer patients, evidence suggests that a concomitant diagnosis of COVID‐19 infection can represent a sentinel event that can change the clinical course, exacerbate complications, and increase the risk of mortality. For this reason, prevention of COVID‐19 infection is critical in this patient population.

## 4 PREVENTION

Public health guidelines were dispatched immediately at the onset of the COVID‐19 pandemic in most developed societies, but supporting information behind many of these interventions due to the novel nature of the disease were not evidence based. Also, the stratification of risk groups and associated prevention measures continue to be controversial given disparate presentations of disease in all groups. For these reasons, appropriate public health intervention and pandemic response continues to be debated in the literature by policymakers and throughout society. Despite this, two solid years of data collected from populations all over the world support the efficacy of two major interventions: mask wearing and vaccination.

Face masks have been deployed previously as a public health measure in societies all over the world. In the United States, masks were required in some locations during the Spanish influenza pandemic with familiar controversy.^8^ But the role of masking has since been studied as a prevention strategy for respiratory pathogens, including evidence from Hong Kong prior to COVID‐19 that showed efficacy in preventing transmission from symptomatic seasonal coronavirus and influenza study subjects.^9^ Since the onset of the COVID‐19 pandemic, mask mandates have been common in many national, regional, and local jurisdictions with observable results, including those documented from Taiwan, Hong Kong, Japan, Singapore, and South Korea.^10^ Furthermore, masks are a resilient intervention that remains intact despite viral evolution resulting in variant strains. Therefore, mask wearing is particularly efficacious in preventing COVID‐19 transmission in high‐risk groups.

The development of vaccines represents one of the single most important public health achievements in modern history. Vaccine‐preventable diseases were a common cause of early and childhood mortality in the United States prior to the development of modern immunization technology. Vaccine science has been applied to expand the prevention of diseases in children and has addressed a number of adult‐onset diseases. The rapid development of the COVID‐19 vaccine represented a major achievement in the interruption of the pandemic, but skeptics question the pace of development and rollout and the evidence that supports safety and efficacy. Also, viral evolution has resulted in a decrease in efficacy in COVID‐19 vaccines reinforced by the sustained circulation of virus in many communities. Further, clinical questions remain regarding safety associated with certain comorbidities and cancer is no exception.

Cancer is an immunocompromising condition as the immune system is often targeted by both the disease state and cancer therapies. Published evidence has shown that active cancer is a risk factor for mortality following infection with COVID‐19. Therefore, current guidelines from the National Comprehensive Cancer Network (NCCN) endorse vaccination for cancer patients along with close contacts and caregivers as the efficacy of vaccination is likely to be lower in cancer patients as compared to the general population. Although the NCCN supports all currently available vaccines, the committee expresses a strong preference for mRNA vaccines. Further, the NCCN stipulates a 3‐month delay in vaccination following hematopoietic cell transplantation or engineered cellular therapy in order to improve vaccine efficacy. These recommendations are in excess of those for the general public, such as delays in vaccination following recent infection or monoclonal antibody treatment that also apply.^11^ It is also important to remain compliant with the booster schedule in order to support sustained immunity. On 29 March 2022, the CDC recommended a fourth booster for select high‐risk groups.^12^

## 5 IMPLICATIONS FOR THE FUTURE

The eradication of viruses on the population level has historically depended upon the achievement of herd immunity acquired through vaccination and infection. However, herd immunity as it relates to COVID‐19 was recently described in a report coauthored by Dr. Anthony Fauci as elusive and likely unattainable. This report describes several distinct challenges, including infection from asymptomatic carriers that thwarts traditional prevention methods. Also, acquired immunity from both vaccination and infection does not appear to be durable notable by the prevalence of breakthrough infections and reinfection. Further, viral evolution has resulted in a consistent supply of variant strains that will continue to challenge both sources of acquired immunity. The authors referenced the Spanish influenza pandemic of 1918, noting that viral decedents continue to circulate and cause infection more than a century later. Another important challenge is managing a coordinated public health response over long periods of time across large geographical regions. Finally, a significant sector of the population has been hostile toward public health measures like vaccination and mask wearing, which has reinforced viral circulation and supported viral evolution. For these reasons, COVID‐19 is likely to become endemic much like influenza. Despite this, concluding comments are optimistic considering important achievements like the widespread availability of testing, proven prevention measures, and the development of effective inpatient and outpatient therapeutics. An important long‐term goal is the development of a universal coronavirus vaccine. This report describes a post‐COVID world in which a low‐level circulation of the virus persists without disrupting daily lives and the health‐care delivery system.^13^

Given the endemic future of COVID‐19, the implications of persistently circulating virus for the treatment of cancer in the future could be significant. First of all, COVID‐19 infection may increase the risk of developing certain types of cancers as the virus targets several proteins involved in the pathology. A recent review discusses the interaction of the COVID‐19 virus with p53 and its pathways, which may lead to oxidative cell and DNA damage. Another potential risk factor is the persistent inhabitation of COVID‐19 in cells following infection, which may lead to prolonged stress and tissue damage.^14^ Evidence also suggests that COVID‐19 infection causes sustained neutrophil dysfunction resulting in susceptibility to both infections and cancer in mild and asymptomatic cases.^15^, ^16^ Other research suggests the presence of a cross‐reactivity mechanism associated with COVID‐19 infection that targets human proteins involved in several malignant processes, including pleuropulmonary blastoma, non‐small cell lung cancer, breast‐invasive ductal carcinoma, multiple human cancers, tumor predisposition syndrome, and mesothelioma.^17^

Another important consideration is the persistence of COVID‐19 symptomology associated with “long COVID” and “post COVID‐19 condition.” “COVID‐long‐haulers” experience a constellation of symptoms according to an often unpredictable pattern for at least 4 months following recovery from active COVID‐19 infection.^18^, ^19^ Long‐COVID symptoms may include chronic cough, fibrotic lung disease, bronchiectasis, fatigue, headaches, myalgia, cognitive symptoms, anxiety and depression, and pulmonary vascular disease.^20^ Population‐level data in the United Kingdom estimates that more than 1 million people were living with long COVID last year.^21^ An American retrospective cohort of 81 million patients identified 273,618 COVID‐19 survivors, 57% of which experienced at least one long COVID symptom in the 6‐month recovery period.^22^ Although little is known about the etiology of long COVID, one theory suggests that COVID long‐haulers experience persistent COVID‐19 infection as evident by viral shedding from the gastrointestinal tract despite negative respiratory tests, which is not uncommon for RNA viruses like human immunodeficiency virus (HIV) and hepatitis C virus (HCV). Subjects evaluated in one long‐COVID study noted lymphocytosis, emphasizing the involvement of specific cells that have known interactions with RNA viruses and cancer cells. Long COVID is believed to produce sustained inflammation and activation of the immune system resulting in clinical symptoms similar to those of autoimmune disorders.^23^, ^24^ Although current data remain unclear regarding the specific risk of long‐term complications of long COVID, including potential increased susceptibility to immune compromising infections and malignancies, the large cohort of COVID‐19 survivors will soon clarify these relationships.

Established cancer patterns of survival and quality of life previously experienced following radiation oncology treatment are likely to be reduced for a number of reasons. First, cancer screening rates remain lower than pre‐pandemic levels coupled with the potential increased risk for developing cancer following COVID‐19 infection. This alone has important implications for cancer care as possible increased prevalence is exacerbated by the late detection of disease. Also, symptoms, interactions, and complications from both COVID‐19 infection and cancer therapies could change the progression of recovery. Further, new data are constantly emerging, which emphasizes the need for treatment standards and guidelines to adapt to the rapid supply of new information. In fact, radiation oncology facilities should actively review and participate in the advancement of knowledge. This process should include expert biomedical scientists who also work as radiation oncology physicists.

As the world reels from a global pandemic, many health‐care and nonhealth‐care fields are being faced with new challenges. Organizations are adjusting visions, goals, interactions, and processes in order to operate in a transformed society. Healthcare has been impacted uniquely as every care center has been touched by new prevention protocols, operations, and new risks and complications in the patients they serve. For cancer care, the sustained impacts of the COVID‐19 pandemic include worsened barriers to cancer screening, the emergence of a new risk for cancer patients, the potential increase in cancer risk for the general population, and the emphasized importance of continuing to accumulate COVID‐19 prevention tools.

## CONFLICT OF INTEREST

The Author has no conflicts of interest to disclose.
